# Delivery Routes for COVID-19 Vaccines

**DOI:** 10.3390/vaccines9050524

**Published:** 2021-05-19

**Authors:** Jang Hyun Park, Heung Kyu Lee

**Affiliations:** Graduate School of Medical Science and Engineering, Korea Advanced Institute of Science and Technology (KAIST), Daejeon 34141, Korea; janghyun.park@kaist.ac.kr

**Keywords:** COVID-19, SARS-CoV-2, vaccine, mucosal vaccine

## Abstract

The novel coronavirus, SARS-CoV-2, which causes COVID-19, has resulted in a pandemic with millions of deaths. To eradicate SARS-CoV-2 and prevent further infections, many vaccine candidates have been developed. These vaccines include not only traditional subunit vaccines and attenuated or inactivated viral vaccines but also nucleic acid and viral vector vaccines. In contrast to the diversity in the platform technology, the delivery of vaccines is limited to intramuscular vaccination. Although intramuscular vaccination is safe and effective, mucosal vaccination could improve the local immune responses that block the spread of pathogens. However, a lack of understanding of mucosal immunity combined with the urgent need for a COVID-19 vaccine has resulted in only intramuscular vaccinations. In this review, we summarize the history of vaccines, current progress in COVID-19 vaccine technology, and the status of intranasal COVID-19 vaccines. Future research should determine the most effective route for vaccine delivery based on the platform and determine the mechanisms that underlie the efficacy of different delivery routes.

## 1. Introduction

Severe acute respiratory syndrome coronavirus 2 (SARS-CoV-2), a novel virus that causes coronavirus disease 2019 (COVID-19), is responsible for a pandemic that has resulted in millions of deaths across the globe. There is an urgent need for effective vaccines to prevent COVID-19 and eradicate SARS-CoV-2, and many companies are developing and testing new vaccines. These include not only traditional vaccines, such as inactivated and attenuated viruses or subunit vaccines, but also new RNA, DNA, and viral vector vaccines [1]. Despite advances in vaccine platform technology, research on the most effective delivery routes for vaccines is limited. Since SARS-CoV-2 infects via the respiratory tract, intranasal vaccination should be effective. However, poor understanding of mucosal vaccines has limited their development to the stage of human clinical trials.

While the angiotensin-converting enzyme (ACE2) receptor for SARS-CoV-2 is found throughout the respiratory tract and in the brain, placenta, and gut, the first line of defense against infection is the nasal epithelium [2]. Intramuscular immunization induces an immune response in the lower respiratory tract (LRT), but not in the upper respiratory tract (URT). In contrast, intranasal immunization provides immunity not only in the URT but also provides systemic immunity [3,4]. Mucosal IgA is known to protect against the shedding of nasal virus early in infection, while the level of systemic IgA is correlated with severe disease. However, mucosal immunity can be difficult to establish because the mucosa is frequently exposed to and becomes tolerant of foreign molecules. Additionally, innate mucosal defense systems such as proteolytic enzymes present a barrier to antigen absorption [5]. For the development of effective mucosal vaccines, we need a better understanding of the mucosal immune environment. Here we review recent progress on delivery routes for COVID-19 vaccines and discuss future perspectives.

## 2. Immune Responses against COVID-19

Understanding the immune responses to SARS-CoV-2 infection is important for designing vaccines for COVID-19. SARS-CoV-2 binds target cells through the ACE2 receptor [6], and then serine protease TMPRRSS2, neuropilin 1, and furins facilitate SARS-CoV-2 infection [7]. As these proteins are broadly expressed in multiple organs including the nasal airway, the lung, and the placenta, SARS-CoV-2 can spread through the body [8]. It is also possible that SARS-CoV-2 can infect immune cells since in vitro infection of monocyte-derived macrophages and dendritic cells (DCs) has been observed and viral RNA was detected in SPP1^+^ macrophages [9].

### 2.1. Innate Immune Response

**Figure 1:**
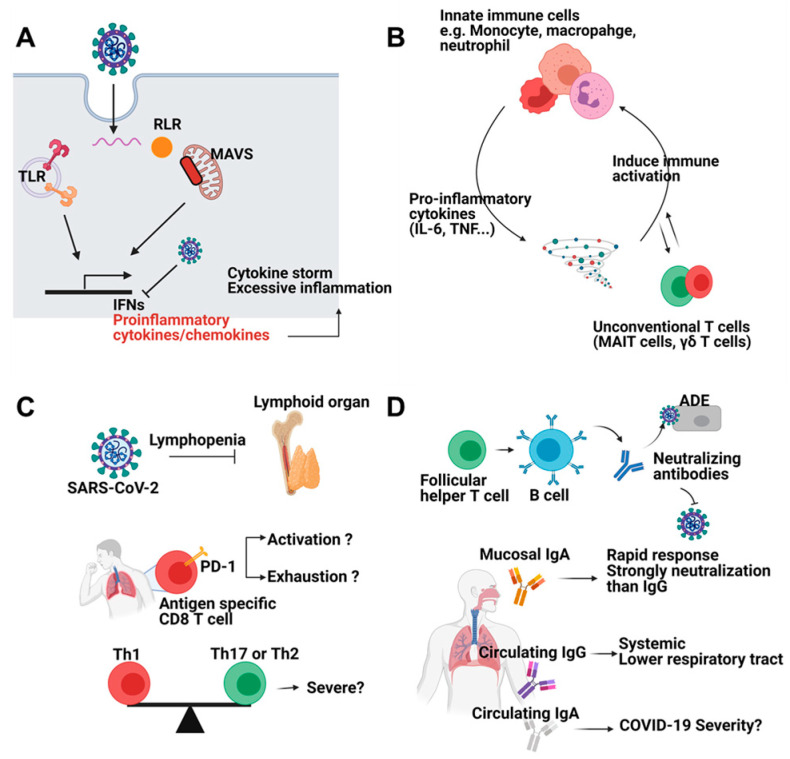
Immune responses against SARS-CoV-2 infection. (**A**) SARS-CoV-2 can be sensed by pattern recognition receptors (PRRs) such as Toll like receptors and RIG-I like receptors. Viral sensing induces interferons and proinflammatory cytokines and chemokines. However, SARS-CoV-2 inhibits induction of interferons. Reduced interferons and excessive proinflammatory cytokines cause cytokine storm and severe inflammation. (**B**) Innate immune cells including monocyte, macrophage, and neutrophils are the main source of proinflammatory cytokines such as IL-6 and TNF. Cytokines further activate immune cells. Unconventional T cells (MAIT cells, γδ T cells) also participate in positive feedback loop of inflammation. (**C**) SARS-CoV-2 infection leads to lymphopenia. CD8 T cells from COVID-19 patients showed exhausted feature. Whether these features represent activation or exhaustion should be addressed. Furthermore, balance among CD4 T cell subsets is a critical factor for disease severity. (**D**) Antibodies from B cells neutralize virus entry. Mucosal IgA is rapidly produced and shows superior neutralizing capacity compared to systemic IgG while circulating IgA is correlated with severe disease symptoms. Circulating IgG systemically protects host from virus. Figure was created by biorender.com (BioRender, Toronto, ON, Canada).

After entry of SARS-CoV-2, multiple pattern recognition receptors (PRRs) including Toll-like receptor 3 (TLR3), TLR7, retinoic acid-inducible gene 1 (RIG-I), and melanoma differentiation-associated gene 5 (MDA5) sense viral infection [7]. PRR recognition triggers phosphorylation of Interferon Regulatory Factor 3 (IRF3) and IRF7 which regulate type I interferon (IFN) and interferon-stimulated genes (ISGs) [10]. SARS-CoV-2-derived nonstructural proteins suppress type I IFN production [11]. As a result, SARS-CoV-2 induces low-level type I and II IFNs alongside high-level induction of proinflammatory cytokines and chemokines [12]. Although a normal immune response protects the host from infection, cytokine release syndrome (CRS), the so-called “cytokine storm”, is frequently observed in patients with severe COVID-19 (Figure 1A) [13].

The main sources of proinflammatory cytokines are monocytes, macrophages, and neutrophils that are recruited and activated by cytokines such as IL-6 and tumor necrosis factor (TNF) or by chemokines. This positive feedback loop increases the severity of the disease [14]. For example, proinflammatory macrophages produce IL-8, which is a chemokine for the recruitment of neutrophils. Neutrophil recruitment correlates with epithelial cell damage and apoptosis which might cause tissue damage and acute respiratory distress syndrome [15]. Programmed death ligand 1 (PD-L1)-expressing neutrophils correlate with severe symptoms while HLA-DR^hi^CD11c^hi^ monocytes show an opposite trend [16]. Mucosal-associated invariant T (MAIT) cells and γδ T cells also correlate with the severity of COVID-19 [17,18]. Although the primary factors leading to uncontrolled inflammation are unknown, the hyperactivated innate immune response is responsible for severe symptoms (Figure 1B).

### 2.2. Adaptive Immune Response

Consistent with other viral infections, T cells and B cells are a critical component of COVID-19, and T cells are phenotypically different depending on the severity of the disease [19]. COVID-19 patients show lymphopenia, with decreased CD4 T cells, CD8 T cells, and B cells, as well as a preference for natural killer cells over CD8 T cells [20,21]. Many viral infections induce transient lymphopenia [22], but SARS-CoV-2 induces a severe, persistent lymphopenia [19] that may be associated with proinflammatory cytokines or activation-induced expression of proapoptotic molecules [20,23]. CD8 T cells from COVID-19 patients express more inhibitory receptors, such as PD-1 and TIM-3, which correlate with terminal differentiation and functional cell exhaustion [23]. SARS-CoV-2-specific CD8 T cells are CD38^+^PD-1^+^ [24]. While it is not clear whether these markers represent cell exhaustion or activation, one study suggests that PD-1-expressing CD8 T cells are functional in COVID-19 patients [25]. CD4 T cells show tendencies similar to CD8 T cells. T helper 1 (Th1) cells correlate with mild disease and CCR6^+^ CD4 T cells correlate strongly with severe disease (Figure 1C) [26,27]. Subsets of T cells, including follicular helper T cell (Tfh) which support B cell responses, correlate with normal antibody-mediated protection [28]. Interestingly, some people who have not been exposed to SARS-CoV-2 have SARS-CoV-2-specific CD4 T cells, which cross-react with common cold viruses [29]. While this protective immunity disappears within 12 months of infection [30], the potential benefits of this cross-reactivity should be examined.

Antibody-mediated neutralization and antibody-dependent cellular cytotoxicity (ADCC) are critical for the clearing of viruses and prevention of infection. Patients with a mild case of COVID-19 have higher levels of immunoglobulins and more activation markers on B cells than patients with severe disease, but there are more CCR2^+^ plasma cells recruited to the lungs of patients with severe disease. Although B cell populations differ depending on the severity of the disease, it is not known whether these changes in cell populations affect the severity of the disease [31]. After acute infection, IgM appears first, followed about 4 weeks later by increasing levels of IgG, which may persist for 16–24 months [32]. Patients with mild-to-moderate symptoms mount a robust antibody response, suggesting that this response provides protective immunity [33]. On the contrary, an opposite result showing asymptomatic patients had lower level of IgG than symptomatic patients was also reported [34]. These contradictory results should be addressed. Unlike to IgG, serum IgA might be associated with severe disease [35,36]. However, IgA appears first after infection and has a stronger neutralization capacity than IgG in COVID-19 [37]. Suboptimal antibody levels can induce antibody-dependent enhancement (ADE), which facilitates viral infection; thus, modulating the antibody response could be beneficial to patients (Figure 1D) [38].

## 3. Current Advances in COVID-19 Vaccines

**Figure 2:**
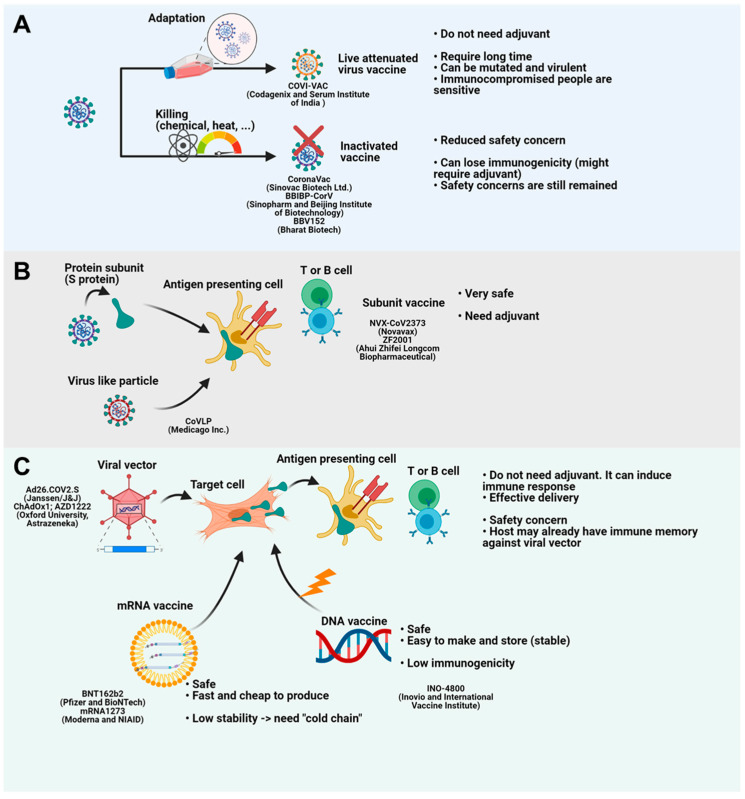
Vaccine platforms for COVID-19. (**A**) Live attenuated vaccines are developed through repeated attenuation. Inactivated vaccines are generated by chemical or heat stress. Live attenuated vaccines do not require adjuvant. However, this technology takes a long time and has safety concerns. Inactivated vaccines have reduced danger compared to attenuated vaccines. However, they need adjuvant because they lose their immunogenicity. (**B**) Protein subunit vaccine or subunit-containing virus like particle (VLP) provides antigen to antigen presenting cells (APCs). APCs present antigen to immune cells and generate immune memory. Although these platforms are safe, they need adjuvant. (**C**) Viral vector vaccines deliver antigen-coding genes to target cells. Infected cells make and give antigen to APCs. Viral vectors do not need adjuvant because the vector itself is danger-associated molecular pattern (DAMP). Delivery is also easy. However, they have safety concerns and vectors can be eradicated by host immune memory if the host has experience of the viral vector. mRNA vaccine and DNA vaccine deliver mRNA and DNA to the target cells. mRNA and DNA generate antigens. While mRNA vaccines are safe, fast and cheap, they need “cold chain” because RNA is unstable. DNA vaccines are stable and safe. However, they are weak to induce proper immunity. Figure was created by biorender.com (BioRender, Toronto, ON, Canada).

More than 100 vaccines for COVID-19 have been or are being tested (Table 1). In contrast to T cells, which frequently target the NSP7, NSP13, and N proteins of SARS-CoV-2 [39], antibodies predominantly target the N protein and the receptor-binding domain (RBD) of the spike (S) protein [40]. Similarly, vaccines typically target the S protein [1]. Traditionally, vaccines are either attenuated or inactivated pathogens or protein subunits from the pathogen. As early as 1880, attenuated pathogen vaccines were developed by Pasteur [1], and attenuated virus vaccines now prevent measles, mumps, and rubella [41]. However, attenuating strains takes years, so this type of vaccine cannot be used to tackle the current pandemic, and there are also safety concerns. Attenuated pathogens can mutate, becoming more virulent, and immunocompromised people might be susceptible to the attenuated strain [1]. Despite their limitations, live attenuated SARS-CoV-2 vaccines for COVID-19 have been developed, and they have the advantage that they do not require adjuvants. COVI-VAC, developed by Codagenix and the Serum Institute of India (Pune, India), is being evaluated in a phase I clinical trial (NCT04619628). Okamura et al. showed that a SARS-CoV-2 vaccine candidate in a Syrian hamster model protected animals from infection and did not produce serious lung lesions (Figure 2A) [42].

To alleviate safety concerns about live attenuated vaccines, pathogens inactivated by heat, radiation, or chemical treatment have been developed as vaccines. However, inactivated pathogens can lose immunogenicity and typically require additional adjuvants [1]. Sinovac Biotech Ltd. developed CoronaVac, an inactivated virus vaccine for COVID-19. CoronaVac, in which the inactivated virus was adsorbed to the adjuvant aluminum hydroxide, induced neutralizing antibodies in mice, rats, and nonhuman primates [55]. A 3 μg dose was found to be safe and immunogenic for people aged 18–59 and for older people in a phase 1/2 clinical trial [43,56]. CoronaVac is now being evaluated in a phase 3 clinical trial (NCT04456595) [57]. BBIBP-CorV, which was developed by the Beijing Institute of Biotechnology and Sinopharm by adsorbing the virus to aluminum hydroxide [58], was found to be immunogenic, well-tolerated, and safe in a phase 1/2 clinical trial [44]. BBIBP-CorV is now being evaluated in a phase 3 clinical trial in multiple countries. Bharat Biotech developed the BBV152 vaccine which is adsorbed to Algel-IMDG (imidazoquinoline molecule chemisorbed on alum). BBV152 is effective in trafficking antigens to the draining lymph node without systemic circulation and has been demonstrated to be immunogenic, well-tolerated, and safe in mice, rats, Syrian hamsters, and nonhuman primates [59,60,61]. A phase 2 clinical trial was successful and BBV152 is now being evaluated in a phase 3 clinical trial (NCT04641481) (Figure 2A) [41].

Protein subunit vaccines contain synthesized or purified viral proteins that are injected and then processed and presented to adaptive immune cells by antigen-presenting cells (APCs). While subunit vaccines are safer than other vaccines, they require adjuvants and booster shots [1]. NVX-CoV2373, which is being developed by Novavax, contains the SARS-CoV-2 S protein within the matrix-M adjuvant. NVX-CoV2373 has been demonstrated to be immunogenic and safe in mice, baboons, and cynomolgus macaques [62,63], and it successfully induced a Th1 response and IgG in a phase 1/2 clinical trial [46]. This vaccine is now being evaluated in a phase 3 clinical trial. Ahui Zhifei Longcom Biopharmaceutical produced the ZF2001 vaccine, a dimeric RBD domain with aluminum hydroxide, which was immunogenic and well-tolerated in a phase 1/2 clinical trial and is being evaluated in a phase 3 clinical trial (NCT04646590) [51]. Medicago Inc. has developed CoVLP, a ‘virus-like particle’ (VLP) comprising empty virus particles containing SARS-CoV-2 S viral surface antigens, which is being evaluated in a phase 2/3 clinical trial (NCT04636697). Medicago also manufactured a plant-based quadrivalent virus-like particle (QVLP) that was successful against influenza infection (Figure 2B) [64].

Viral vectors including adenoviruses, modified vaccinia Ankara (MVA), and vesicular stomatitis virus (VSV) have been used successfully to produce vaccines against pathogens such as the Ebola virus [1]. However, the host may already have immunity against some viral vectors, which must be considered in determining the prime and boost doses. CanSino Biologics made a recombinant adenovirus type-5 (Ad5) vector vaccine expressing the S protein, Ad5-nCoV, that was fully immunogenic, and a single dose induced a significant immune response [49,65]. Sputnik V (Gam-COVID-Vac) is a combination vector vaccine of rAd26 and rAd5 containing the gene for the S protein, which had an efficacy rate of 91.6% in a preliminary report of a phase 3 clinical trial [49].

Ad5 is the most common adenovirus type that infects humans, and many people might have contracted the virus and may be immune to it; however, Ad26 is less common [1]. The Ad26.COV2.S vector containing the gene for the S protein, developed by Janssen, produced a durable immune response. A single dose of Ad26.COV2.S induced a humoral immune response in more than 90% of participants [66]. Ad26.COV2.S is being evaluated in a phase 2/3 clinical trial (NCT04614948). In phase 3 trial, single shot of Ad26.COV2.S was safe and effective [49]. Ad26.COV2.S is currently in use as the “J&J/Janssen” vaccine. To avoid pre-existing immunity, AstraZeneca and Oxford University used a chimpanzee adenovirus to deliver the gene for the S protein (ChAdOx1; AZD1222). AZD1222 was immunogenic and well-tolerated; however, older people showed greater tolerance than younger people in a phase 2/3 clinical trial [67]. A single dose of AZD1222 fully induced the T cell response [68], but after a second dose, the antibody response was stronger [69]. AZD1222 is currently in use as the “AstraZeneca” vaccine (Figure 2C).

The COVID-19 pandemic has led to the rapid development of DNA and RNA vaccines, which are currently being tested and used. Antigen-coding DNA is delivered intradermally or intramuscularly where local myocytes or nascent cells take up the DNA and synthesize the antigen. The antigen is taken up by antigen-presenting cells and then presented to adaptive immune cells. For effective delivery, DNA vaccines need to be electroporated into cells [1]. INO-4800, which contains DNA coding for the S protein of SARS-CoV-2 has been developed by Inovio and International Vaccine Institute. INO-4800 is delivered intradermally and a small electric pulse is applied to the skin leading to cellular uptake of the DNA. The DNA vaccine was immunogenic and well-tolerated in 100% of vaccinated subjects in a phase 1 clinical trial [52]. INO-4800 is being evaluated in an ongoing phase 2/3 clinical trial (INNOVATE; NCT04642638) (Figure 2C) [1].

RNA vaccines have been at the forefront of rapid vaccine development due to COVID-19. Pfizer and BioNTech produced BNT162b2, a vaccine formulated using mRNA for the S protein encased in lipid nanoparticles. Two doses of BNT162b2 showed 95% protection in a phase 2 clinical trial [53]. Although the phase 3 clinical trial (NCT04713553) is still recruiting, BNT162b2 was the first mRNA COVID-19 vaccine approved for use [70]. BNT162b2 is currently in use as the “Pfizer/BioNTech” COVID-19 vaccine. Interestingly, 12–37 days after the first dose of the vaccine, the viral loads of patients were significantly decreased [71]. The second mRNA vaccine is mRNA-1273, developed by Moderna and NIAID, which also contains mRNA coding for the S protein inside lipid nanoparticles. A preliminary report of a phase 2 trial showed that mRNA-1273 induced an immune response against SARS-CoV-2 [72]. Result of phase 3 clinical trial of mRNA-1273 showed 94.1% efficacy at preventing COVID-19 disease [50]. After a second dose of mRNA-1273, antibodies persisted for 6 months [73]. mRNA-1273 is currently in use as the “Moderna” vaccine (Figure 2C).

Several other vaccines are under development or still in the preclinical trial or clinical trial stage of development. Several vaccines are already approved and in current use.

## 4. Delivery Route of Vaccines

Most vaccines are injected intramuscularly [1] because the traditional subcutaneous route for vaccines that include an aluminum salt adjuvant resulted in severe adverse effects [74]. A clinical trial for a diphtheria toxin (DT) vaccine with a booster shot demonstrated that an intramuscular vaccination gave significantly fewer adverse effects than a subcutaneous injection [75]. Intramuscular injections of the H3N2 and H1N1 influenza vaccines were more immunogenic than a subcutaneous injection [76]. One vaccine study in rhesus macaques using the HIV-1 envelope glycoprotein (Env) in synthetic liposomes gave comparable immune responses for intramuscular and subcutaneous delivery [77]. The authors of a systematic review of delivery methods recommended intradermal rather than subcutaneous or intramuscular injection, when appropriate, because an intradermal injection requires a lower dose of the vaccine [78].

The route of immunization may induce different mechanisms of protection. An aerosol vaccination of the DNA prime-Ad5 booster vaccine for Simian immunodeficiency virus (SIV) antigens induced IgA-driven neutrophil-mediated phagocytosis, while intramuscular injection induced IgG-driven antibody-dependent monocyte-mediated phagocytosis [79]. However, these varied results came from different vaccine platforms suggesting that the efficacy of the vaccination route might be related to the platform technology, such as mRNA, DNA, or an adenoviral vector vaccine.

The discovery of tissue-resident memory T cells (Trm) showed that memory T cells residing in nonlymphoid tissue can respond immediately to pathogens [80,81]. The prime and pull strategy, which comprises a conventional parenteral vaccination and recruitment of activated T cells to the local area, establishes long-lasting local immune memory and effective protection against vaginal herpes simplex virus type 2 (HSV-2) infection [82]. Enhanced Trm accumulation requires repeated local antigen recognition [83]. Although circulating IgG can protect the host from mucosal infection, local IgA from plasma cells inhibits infection of local target cells. After influenza infection, local plasma cells secrete IgA to protect type I mucosal cells of the upper respiratory tract while IgG protects lung cells [84]. CD4 Trm cells recruit circulating memory B cells to secrete IgG and IgA in the type II mucosa [85]. These results suggest that local vaccination might be more effective than peripheral vaccination for mucosal infection.

**Figure 3:**
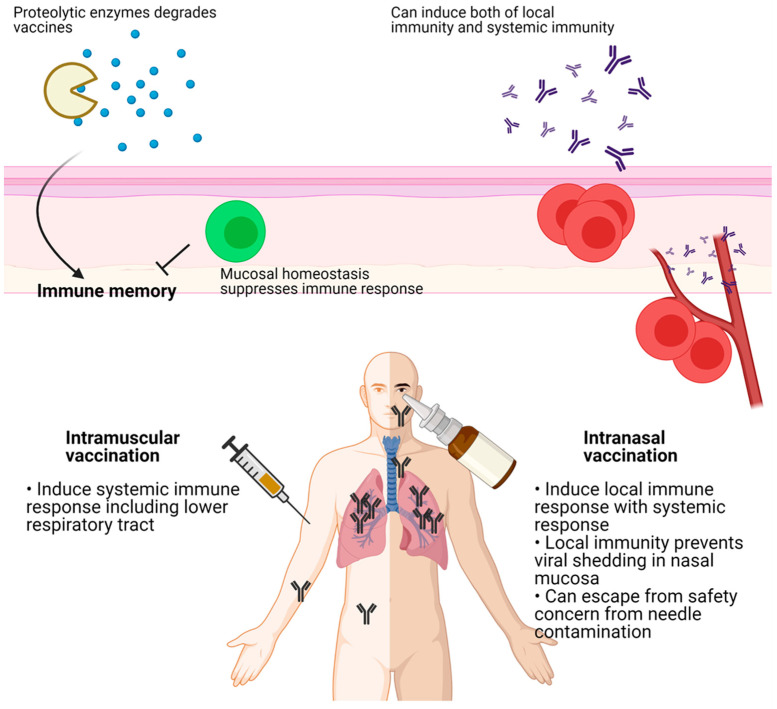
Intramuscular vaccination induces systemic immune response including lower respiratory tract (LRT), but not upper respiratory tract (URT). In contrary, intranasal vaccination induces local immune response in LRT with systemic immunity. Local immunity prevents nasal shedding of viruses which is critical for herd immunity. In the mucosal area, delivered antigens can be degraded by proteolytic enzymes. Mucosal homeostasis inhibits immune response against vaccines. Figure was created by biorender.com (BioRender, Toronto, ON, Canada).

In addition to efficacy, mucosal vaccination is safer than peripheral vaccination because it avoids possible blood-borne infections from contaminated needles. While mucosal vaccination can induce a systemic immune response [86], the development of mucosal vaccines is difficult because little is known about mucosal immunity. The mucosal vaccine response could be attenuated since the mucosa maintains homeostasis in response to foreign invasion, and absorption of antigens through the mucosal barrier might be difficult as the antigens could be destroyed by proteolytic enzymes (Figure 3) [87]. Additionally, many vaccine platforms such as subunit and inactivated virus vaccines need adjuvants for an effective immune response. However, common adjuvants, including alum, could not induce the IgA and IgG class-switching and the recruitment of T and B cells to the mucosal area. Thus, infectious viral vectors that can trigger pathogen-associated molecular patterns (PAMPs) might be more suitable for mucosal vaccines [88].

## 5. Intranasal Vaccination of COVID-19 Vaccines

Currently, all vaccines for COVID-19 are injected intramuscularly. However, because SARS-CoV-2 mainly infects via the upper respiratory tract, the microenvironment of the nasal passage is important for immunity. For example, intranasal immunization of mice using the N protein resulted in a higher T cell response in lung bronchoalveolar lavage (BAL) than did intramuscular injection, while responses in the trachea and the oral cavity were similar. In contrast, T cell responses in the spleen, inguinal lymph nodes, and the brain were lower with intranasal immunization than with intramuscular injection [89]. This suggests that the route of immunization affects the immune responses of multiple organs, not only the target organ.

Several experiments using intranasal vaccinations have given generally favorable results. Intranasal vaccination using SARS-CoV induced Trm cells in the lungs while subcutaneous injection established memory T cells in the spleen, and airway Trm cells were important for protection from SARS-CoV infection [90]. Intramuscular ChAdOx1 injection in rhesus macaques reduced viral load in the bronchoalveolar lavage fluid (BALF) and prevented pneumonia, but did not prevent nasal shedding of the virus [3]. Intranasal ChAd-SARS-CoV-2-S (based on the simian Ad-36 vector) shows superior protection and mucosal immunity compared to the parenteral route in a murine model [91] and prevented viral shedding in rhesus macaques [4]. Intranasal and subcutaneous Ad5.SARS-CoV-2-S1 vaccination induced robust humoral and cellular immune responses in a murine model [92]. Administration of Ad5-S-nb2 in the nasal cavity also induced a systemic and a local antibody response that protected macaques [93]. Local administration of a lentivirus vector induced an IgA response that protected mice from SARS-CoV-2 [94]. Intranasal immunization with inactivated SARS-CoV with adjuvants was sufficient to induce local and serum antibodies in mice [95]. Similarly, nasal administration of a recombinant RBD vaccine with alum induced protective immunity against SARS-CoV-2 in mice [96]. These results suggest that intranasal administration of vaccines protects the host from SARS-CoV-2 infection and that local vaccination was sufficient to induce systemic humoral immune responses.

However, other experiments gave mixed results from intranasal vaccinations. Intranasal injection of human neutralizing antibodies (HuNAbs) and a DNA vaccine did not reduce the viral load in the nasal turbinate, but the lung was protected [97]. Intramuscular delivery of VSV-SARS2-EBOV vaccine demonstrated rapid protection compared to an intranasal injection [98]. However, these results contradict previous studies. While it is not yet clear whether intranasal vaccination is superior to intramuscular vaccination, it is evident that intranasal vaccination protects the host from infection. The efficacy of an intranasal vaccination may depend on the dosage or the vaccine platform. However, because nasal immunization can provide sterilizing immunity, preventing interhuman transmission, it might be an effective route to herd immunity [5]. While several clinical trials of intranasal vaccines, including AdCOVID (altimmune; Gaitersburg, USA), are currently in progress [1], further studies are needed to determine the most effective route for immunization.

## 6. Conclusions

The rapid development of vaccine technology has led to several vaccines for COVID-19. Some of the vaccines that have been approved for emergency use include mRNA-1273 (Moderna), AZD1222 (AstraZeneca), and Ad26.COV2.S (J&J/Janssen), which are delivered intramuscularly, and the results of their global use will provide important information on their safety and efficacy. As the local immune microenvironment is important for protection against infection and for preventing viral shedding, intranasal immunization is expected to be more effective than intramuscular immunization. While research has shown that in some cases intranasal immunization is better than an intramuscular injection, there are also contradictory results. Depending on the vaccine platform, the efficacy of intranasal immunization may differ. For example, a live viral vector may be more effective in inducing an immune response because it does not require an adjuvant. Since we do not fully understand mucosal immunity, future studies should focus on how the vaccine platform affects the mucosal immune response.

## Figures and Tables

**Table 1:** Current advances in COVID-19 vaccines.

Name	Company	Route	Technology	Clinical Trial	Approved	Grade 3-4 Side Effects
COVI-VAC	Codagenix (NewYork, USA) Serum Institute (Pune, India)	Intranasal	Live-attenuated virus	Phase I clinical trial (NCT04619628)	No	
CoronaVac	Sinovac Biotech Ltd. (Beijing, China)	Intramuscular	Inactivated virus	Phase III clinical trial (NCT04800133, NCT04651790, NCT04456595, NCT04508075, NCT04582344, NCT04617483, PHRR210210-003308)	Yes	Wu et al. [43] phase 1/2 trial No
BBIBP-CorV	Sinopharm Beijing Institute of Biotechnology (Beijing, China)	Intramuscular	Inactivated virus	Phase III clinical trial (NCT04510207, ChiCTR2000034780, NCT04612972, NCT04560881)	Yes	Xia et al. [44] phase 1/2 trial, No
BBV152 (Covaxin)	Bharat Biotech (Hyderabad, India)	Intramuscular	Inactivated virus	Phase III clinical trial (NCT04641481)	Yes	Ella et al. [45]. phase 2 trial, days 0–7, 28–35 No
NVX-CoV2373	Novavax (Gaithersburg, USA)	Intramuscular	Protein subunit	Phase III clinical trial (NCT04583995, NCT04611802)	No	Keech et al. [46]. phase 1–2 trial 2% (severe adverse events) for groups D and E
ZF2001	Ahui Zhifei Longcom Biopharmaceutical (Hefei, China)	Intramuscular	Protein subunit	Phase III clinical trial (NCT04646590)	Yes	Yang et al. [47]. phase 1 and 2 trial 10% (grade 3 or worse) for 50 μg group
CoVLP	Medicago Inc. (Quebec, ON, Canada)	Intramuscular	Virus-like particle	Phase III clinical trial (NCT04636697)	No	
Ad5-nCoV	Cansino Biologics (Tianjin, China)	Intramuscular	Adenovirus vector (Ad5)	Phase III clinical trial (NCT04526990, NCT04540419)	Yes	Zhu et al. [48] phase 2 trial 9% (grade 3) 1 × 10^11^ group, 1% (grade 3) 5 × 10^10^ group
Sputnik V	Gamaleya (Moscow, Russia)	Intramuscular	Adenovirus vector (Ad5 + Ad26)	Phase III clinical trial (NCT04640233, NCT04642339, NCT04656613, NCT04741061, NCT04564716, NCT4530396)	Yes	Logunov et al. [49]. phase 3 trial 0.38% (grade 3)
Ad26.COV2.S	Johnson and Johnson (Janssen) (Beerse, Belgium)	Intramuscular	Adenovirus vector (Ad26)	Phase III clinical trial (NCT04838795, NCT04505722, NCT04614948)	Yes	Sadoff et al. [50]. phase 3 trial 0.4% (serious adverse events)
AZD1222	AstraZeneca (Cambridge, UK), Oxford university (Oxford, UK)	Intramuscular	ChAdOx1	Phase III clinical trial (NCT04864561, NCT04800133, NCT04536051, NCT04516746, NCT04400838, NCT04540393)	Yes	Voysey et al. [51]. pooled four trials 0.7% (serious adverse events)
INO-4800	Inovio (Plymouth Meeting, USA), International vaccine institute (Seoul, South Korea)	Intradermal	DNA vaccine	Phase III clinical trial (NCT04642638)	No	Tebas et al. [52]. phase 1 trial No
BNT162b2	Pfizer (New York, USA), BioNTech (Mainz, Germany)	Intramuscular	mRNA vaccine	Phase III clinical trial (NCT04368728, NCT04805125, NCT04800133, NCT04816669, NCT04713553, NCT04754594)	Yes	Polack et al. [53]. phase 2/3 trial 4/43,252 (serious adverse events), 2/43,252 (died)
mRNA-1273	Moderna (Cambridge, USA), NIAID (North Bathesda, USA)	Intramuscular	mRNA vaccine	Phase III clinical trial (NCT04860297, NCT04806113, NCT04649151, NCT04470427, NCT04796896, NCT04811664, NCT04805125)	Yes	Baden et al. [54]. phase 3 trial 1.5% (grade 3)

## Data Availability

No new data was generated.
